# Supplementary material for: Impact of food availability and predator presence on patterns of landscape partitioning among neighbouring Guinea baboon (Papio papio) parties
Source: Mov Ecol. 2025 Feb 22;13:9. doi: 10.1186/s40462-025-00534-9 (PMC11847332; doi:10.1186/s40462-025-00534-9)
Supplement: Supplementary file 1 — Additional file1 . [file 40462_2025_534_MOESM1_ESM.pdf]

Supplementary Information

Impact of food availability and predator presence on patterns of landscape partitioning among neighbouring Guinea baboon (*Papio papio*) parties

Lisa Ohrndorf, Roger Mundry, Jörg Beckmann, Julia Fischer, Dietmar Zinner

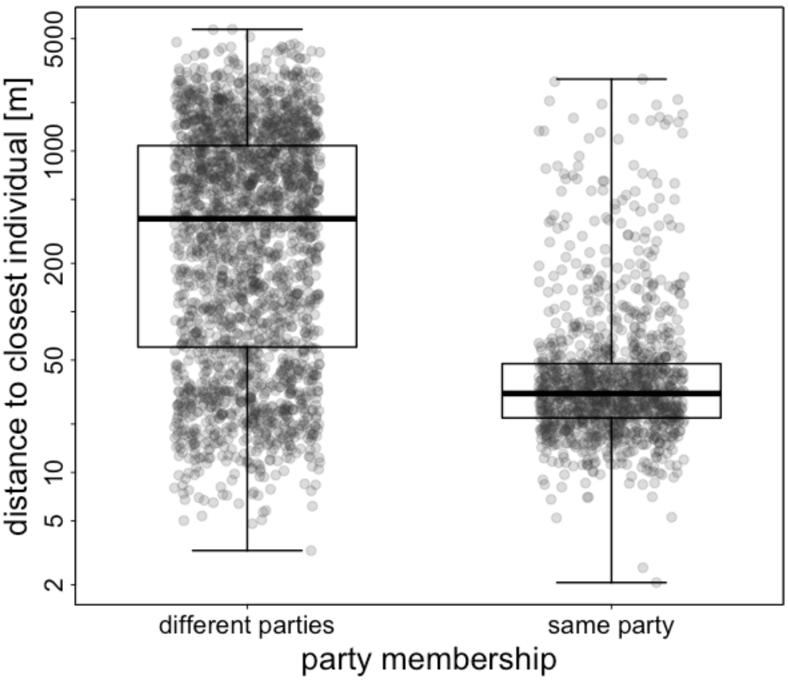

Fig. S1: Average minimum distances between individuals of the same parties compared to individuals of different parties that were collared from 2010 to 2012. Boxplots depict the median (black line) and IQR with the lower (25%) and upper (75%) quartile. Whiskers represent the 2.5th and 97.5th percentiles. The distance to the closest party is depicted on a log-scale for visual clarity.

Table S1: Tree species subject to phenological monitoring and respective plant parts that baboons fed on more than once according to Klapproth et al. (unpublished data) (1 = occasional food resource, 2 = common food resource, 3 = key food resource).

Family	Species	used	parts eaten
Anacardiaceae	*Lannea microcarpa*	3	fruits
Annonaceae	*Hexalobus monopetalus*	2	fruits, flowers
	Spondias mombin	2	fruits
Apocynaceae	*Saba senegalensis*	1	fruits
Arecaceae	*Borassus akeassii*	3	fruits, fibre, petiole
Bombacaceae	*Bombax costatum*	2	seeds
Caesalpinioideae	*Cordyla pinnata*	2	fruits
	Piliostigma spp.	2	pods, seeds
	P. reticulatum		
	P. thoningii		
	Tamarindus indica	2	fruits
Capparaceae	*Capparis tomentosa*	1	seeds
Combretaceae	*Combretum* spp.	2	seeds
	C. glutinosum		
	C. micranthum		
	Terminalia macroptera	2	seeds, bark
Ebenaceae	*Diospyros mespiliformes*	1	fruits
Loganiaceae	*Strychnos spinosa*	2	fruits
Mimosiodeae	*Mimosa pigra*	2	pods, seeds
	Acacia spp.	2	pods, seeds, flowers
	A. macrostachya		
	A. seyal		
	A. sieberiana		
	Dicrostachys cinera	2	pods, seeds
	Parkia biglobosa	2	fruits
Moraceae	*Ficus ingens*	2	fruits
Papilionoideae	*Pterocarpus erinaceus*	3	seeds, bark
Rhamnaceae	*Ziziphus* spp.	2	fruits
	Z. mauretania		
	Z. mucronata		
Rubiaceae	*Sarcocephalus latifolius*	2	fruits
Tiliaceae	*Grewia* spp.	2	fruits
	Grewia bicolor		
	Grewia lasiodiscus		
Ulmaceae	*Celtis integrifolia*	2	fruits
Verbenaceae	*Vitex madiensis*	1	fruits

Table S2: Overlap between home ranges (KDE, 95% contour level) of parties observed in 2010. Values represent the proportion of overlap (0 = no overlap, 1= full overlap). The extent of the overlap is indicated as a colour gradient from green (low overlap) to yellow (high overlap).

	P4	P5	P6	P9
P4		0.68	0.72	0.92
P5	0.95		1.00	0.94
P6	0.93	0.92		0.92
P9	0.82	0.64	0.69	

Table S3: Overlap between home ranges (KDE, 95% contour level) of parties observed from 2011 to 2012. Values represent the proportion of overlap (0 = no overlap, 1= full overlap). The extent of the overlap is indicated as a colour gradient from green (low overlap) to yellow (high overlap).

	P4	P5	P6	P9
P4		0.83	0.81	0.81
P5	0.88		0.96	0.73
P6	0.92	0.96		0.82
P9	0.95	0.80	0.77	

Table S4: Overlap between core areas (KDE, 50% contour level) of parties observed in 2010. Values represent the proportion of overlap (0 = no overlap, 1= full overlap). The extent of the overlap is indicated as a colour gradient from green (low overlap) to yellow (high overlap).

	P4	P5	P6	P9
P4		0.71	0.80	0.99
P5	0.63		0.98	0.65
P6	0.59	0.86		0.61
P9	0.85	0.67	0.76	

Table S5: Overlap between core areas (KDE, 50% contour level) of parties observed from 2011 and 2012. Values represent the proportion of overlap (0 = no overlap, 1= full overlap). The extent of the overlap is indicated as a colour gradient from green (low overlap) to yellow (high overlap).

	P4	P5	P6	P9
P4		0.80	0.72	0.87
P5	0.51		0.97	0.61
P6	0.56	0.93		0.59
P9	0.88	0.83	0.68	

Table S6: Model results on spatiotemporal landscape partitioning between neighbouring parties in response to food availability and number of predator encounters within 7 days (estimates, standard errors, 95% credible intervals, Rhat, and Bulk and Tail Effective Sample Sizes).

Term	Estimate	Est.Error	CI_{lower}	CI_{upper}	Rhat	Bulk_ESS	Tail_ESS
Intercept	5.15	0.71	3.67	6.49	1.00	1221	1826
pred.enc.7	-0.00	0.01	-0.02	0.02	1.00	2909	2878
food.score	0.19	0.52	-0.80	1.30	1.00	2755	2684

Table S7: Model results on spatiotemporal landscape partitioning between neighbouring parties in response to food availability and number of predator encounters within 30 days (estimates, standard errors, 95% credible intervals, Rhat, and Bulk and Tail Effective Sample Sizes).

Term	Estimate	Est.Error	CI_{lower}	CI_{upper}	Rhat	Bulk_ESS	Tail_ESS
Intercept	5.49	0.84	3.79	7.14	1.00	975	1470
pred.enc.30	-0.01	0.01	-0.02	0.01	1.00	1555	1966
food.score	0.35	0.53	-0.64	1.43	1.00	1866	1944

Table S8: Model results on spatiotemporal landscape partitioning between neighbouring parties in response to food availability and number of predator encounters within two days (estimates, standard errors, 95% credible intervals, Rhat, and Bulk and Tail Effective Sample Sizes).

Term	Estimate	Est.Error	CI_{lower}	CI_{upper}	Rhat	Bulk_ESS	Tail_ESS
Intercept	5.00	0.63	3.63	6.19	1.01	731	925
pred.enc.2	0.03	0.03	0.03	0.08	1.00	1391	1667
food.score	0.13	0.54	0.54	1.24	1.00	1589	1797

Table S9: Model results on spatiotemporal landscape partitioning between neighbouring parties **at noon** in response to food availability and number of predator encounters within two weeks (estimates, standard errors, 95% credible intervals, Rhat, and Bulk and Tail Effective Sample Sizes).

Term	Estimate	Est.Error	CI_{lower}	CI_{upper}	Rhat	Bulk_ESS	Tail_ESS
Intercept	4.36	0.72	2.84	5.79	1.00	1342	1948
pred.enc.14	0.01	0.01	-0.01	0.04	1.00	3795	2875
food.score	-0.30	0.70	-1.66	1.08	1.00	3370	2584

Table S10: Model results on spatiotemporal landscape partitioning between neighbouring parties **at noon** in response to food availability and number of predator encounters within 7 days (estimates, standard errors, 95% credible intervals, Rhat, and Bulk and Tail Effective Sample Sizes).

Term	Estimate	Est.Error	CI_{lower}	CI_{upper}	Rhat	Bulk_ESS	Tail_ESS
Intercept	4.37	0.74	2.89	5.80	1.00	1161	1705
pred.enc.7	0.01	0.01	-0.01	0.04	1.00	4081	2944
food.score	-0.31	0.66	-1.69	0.94	1.00	3747	3000

Table S11: Model results on spatiotemporal landscape partitioning between neighbouring parties **at noon** in response to food availability and number of predator encounters within 30 days (estimates, standard errors, 95% credible intervals, Rhat, and Bulk and Tail Effective Sample Sizes).

Term	Estimate	Est.Error	CI_{lower}	CI_{upper}	Rhat	Bulk_ESS	Tail_ESS
Intercept	4.68	0.84	3.01	6.37	1.00	1310	2200
pred.enc.30	-0.00	0.01	-0.02	0.01	1.00	2306	2326
food.score	-0.03	0.81	-1.70	1.58	1.00	2594	2165

Table S12: Model results on spatiotemporal landscape partitioning between neighbouring parties **at noon** in response to food availability and number of predator encounters within 2 days (estimates, standard errors, 95% credible intervals, Rhat, and Bulk and Tail Effective Sample Sizes).

Term	Estimate	Est.Error	CI_{lower}	CI_{upper}	Rhat	Bulk_ESS	Tail_ESS
Intercept	4.46	0.68	3.06	5.86	1.01	1291	1974
pred.enc.2	0.04	0.03	-0.02	0.09	1.00	3587	2268
food.score	-0.25	0.69	-1.60	1.13	1.00	3295	2414
